# Incidence and risk of fatal adverse events in cancer patients treated with HER2-targeted antibody-drug conjugates: a systematic review and meta-analysis of randomized controlled trials

**DOI:** 10.1186/s12885-023-11250-1

**Published:** 2023-10-10

**Authors:** Zhiwen Fu, Chen Gao, Jiyi Xie, Cong Zhang, Shijun Li, Ming Gu, Chen Shi

**Affiliations:** 1 Department of Pharmacy, Union Hospital, Tongji Medical College, Huazhong University of Science and Technology, Wuhan, Hubei Province China; 2 Hubei Province Clinical Research Center for Precision Medicine for Critical Illness, 1277 Jiefang Avenue, Jianghan District, Wuhan, Hubei Province 430000 China

**Keywords:** HER2-targeted ADC, Fatal adverse events, Incidence and risk, Systematic review, Meta-analysis

## Abstract

**Objective:**

Antibody-drug conjugates (ADCs) that target human epidermal growth factor receptor 2 (HER2) are leading a new era of targeted cancer therapy. These drugs have also been associated with several fatal adverse events, such as pneumonia, interstitial lung disease, and infection. We performed a meta-analysis of randomized controlled trials (RCTs) to estimate the incidence and risk of fatal adverse events in cancer patients treated with HER2-targeted ADCs.

**Methods:**

We performed a systematic search in Embase, PubMed, Web of Science, and Scopus databases from inception to February 1, 2022, and the last search was updated to July 1, 2023. The eligible studies for inclusion in our analysis were limited to RCTs of HER2-targeted ADCs that were approved by the US Food and Drug Administration and examined on cancer patients with available data on fatal adverse events. The protocol for this study was registered in PROSPERO (No. CRD42022331627).

**Results:**

Fifteen studies (13 RCTs) involving 7,277 patients were finally included for meta-analysis. Of these patients, 4,246 received HER2-targeted ADCs and 3,481 received the control treatment. The data were combined using Bayesian hierarchical modeling, which allowed for the estimation of the mean incidence of fatal adverse events to be 0.78% (95% CrI: 0.28-1.37%, τ = 0.006) for the patients treated with HER2-targeted ADCs. The relative risk was 0.80 (95% CrI, 0.5–1.26, τ = 0.17) compared to control patients. Among 43 reported deaths caused by HER2-targeted ADCs, the most common fatal adverse event was respiratory toxicity, including pneumonia, pneumonitis, and interstitial lung disease. On subgroup analysis, no difference in the risk of fatal adverse events was found between different HER2-targeted ADCs or cancer types.

**Conclusion:**

Our findings suggest that the risk of fatal adverse events with HER2-targeted ADCs may be lower compared to standard control therapies in cancer patients, and there is no significant difference in risk observed between different HER2-targeted ADCs or cancer types. However, the most common fatal adverse event was respiratory toxicity, suggesting that cancer patients who use the above drugs should strengthen respiratory system monitoring and take preventive measures in some severe cases.

**Supplementary Information:**

The online version contains supplementary material available at 10.1186/s12885-023-11250-1.

## Introduction

Human epidermal growth factor receptor 2 (HER2) plays a crucial role in tumor growth, invasion, and development [1, 2]. Extensive studies have shown that HER2 expression is closely related to the occurrence of various tumors and is one of the most important targets for developing anticancer therapies [3, 4]. Antibody-drug conjugate (ADC), which is composed of a monoclonal antibody linked to a cytotoxic agent, is revolutionizing targeted cancer therapy [5]. HER2, as a classic tumor target, has become an ideal target for the development of ADC drugs due to its high specific expression in tumor tissue and its high efficiency in mediating the endocytosis of ADC drugs. To date, the US Food and Drug Administration (FDA) has approved two ADC drugs (T-DM1 and T-DXd), and over 60 HER2-targeted ADC candidates are currently undergoing clinical trials [6].

HER2-targeted ADCs have shown excellent efficacy and have had their indications expanded on the strength of their ingenious design of the molecular structure that delivers cytotoxic drugs specifically to cancer cells [7–9]. However, fatal adverse events have been reported with HER2-targeted ADCs due to undesired uptake in healthy cells. Some of these adverse events can be life-threatening, such as pneumonitis, hematotoxicity, cardiotoxicity, and hepatotoxicity [10–12]. The occurrence of fatal adverse events is difficult to avoid during anticancer treatments and causes great harm to patients and their families. In order to improve the treatment compliance of patients and ensure that patients can continue to benefit from the treatment of HER2-targeted ADCs, it is particularly important to clearly understand the profile of fatal adverse events and timely develop management measures. And the analysis of fatal adverse events will help to improve the guidelines and provide guidance for better guiding the clinical application of HER2-targeted ADC drugs.

Multiple clinical trials have been concerned with the fatal adverse events caused by HER2-targeted ADCs, but the limited number of patients in each trial has left the overall incidence and risk of such events unclear. Therefore, in this study, we conducted a systematic review and meta-analysis of the mortality profile of HER2-targeted ADCs. Utilizing a Bayesian hierarchical modeling approach, we quantitatively combined data from randomized controlled trials (RCTs) to address the incidence and risk of fatal adverse events in cancer patients treated with HER2-targeted ADCs. We aim to provide clinicians with a reference to use HER2-targeted ADCs appropriately and manage potential fatal adverse events related to these drugs.

## Methods

### Search methods and study selection

The present systematic review was conducted according to the Preferred Reporting Items for Systematic Reviews and Meta-analyses (PRISMA) guideline (**eTable 1** in the Supplement) [13, 14]. The protocol was registered in PROSPERO (No. CRD42022331627). We systematically searched the PubMed, Embase, Web of Science, and Scopus databases from inception to February 1, 2022, and updated the last search to July 1, 2023. The keywords, including “HER2 ADC”, “trastuzumab emtansine (T-DM1)”, and “trastuzumab deruxtecan (T-Dxd)”, are employed and full search strategy is detailed in **eTable 2** in Supplement. We also manually screened reference lists from relevant review articles to supplement the search. This study excluded non-randomized trials, editorials, correspondences, and reviews. We included only prospective RCTs of HER2-targeted ADC agents in the treatment of cancer patients. The inclusion criteria were based on the PICO-framework. In detail, Population (P): cancer patients; Intervention (I): treatments by HER2-targeted ADC agents. Comparison (C): The other therapeutics. Outcomes (O): any fatal adverse events. When publications reported the same trial, the most recent one was included. Two reviewers (JY Xie and C Zhang) were independently responsible for screening and reviewing the included literature and ZW Fu was involved to discuss and reach a consensus when there is a controversy.

### Data extraction

The primary outcome was the mortality profile of HER2-targeted ADCs, including the incidence and relative risk (RR) of the fatal adverse event, which was defined as deaths caused most likely by the treated ADC drugs. Baseline information, such as authors, publication years, trial names, NCT numbers, trial phases, cancer types, and the used ADC therapies, were also extracted from each included trial.

### Statistical analysis

The incidences of fatal adverse events were calculated by dividing the number of patients who experienced adverse events by the total number of patients. Since the incidence of fatal adverse events is typically low and some studies included zero events in the treatment and/or control groups, the variance for such studies approaches zero. Consequently, the weight of these studies would be overestimated in a classic meta-analysis. To address this issue, we utilized a Bayesian hierarchical modeling strategy to conduct the meta-analysis of fatal adverse events in this study.

For the *i*th study which reported the dichotomous outcomes, the number of patients with reported any adverse events in the *i*th study followed the binomial distribution: ri ~ binomial (𝑛𝑖, 𝑝𝑖), where 𝑛𝑖 was the total number of investigated populations and 𝑝𝑖 was the incidence of adverse events for the *i*th study. The logit transformation of 𝑝𝑖 followed a normal distribution among studies: 𝜃𝑖 = logit(𝑝𝑖) ~ normal (𝜇, 𝜎2), where 𝜇 was the mean of logit(𝑝𝑖) and 𝜎2 was the between-study variance. Then we could estimate the pooled incidence of adverse events and the corresponding 95% credible interval (CrI) through retransform: Incidence = exp (𝜇)/(1 + 𝑒𝑥𝑝 (𝜇)).

Bayesian inference was utilized to estimate the pooled effect sizes through the combination of prior information and observed sampling distribution [15]. The Bayesian random effects model was applied to generate the estimates of the overall incidence and relative risk compared to the control arm, along with a 95% credible interval (CrI). The CrI represented the 2.5–97.5 percentiles of the posterior distribution of the estimation. For the mean parameters of normal distributions, a proper prior distribution with mean = 0 and sd = 4 was proposed. The between-study variances were assigned weakly informative normal prior distributions with the mode at 0 and the scale at 1. The posterior distribution of interest outcomes was estimated using the Markov Chain Monte Carlo (MCMC) algorithm and Gibbs sampling in all Bayesian hierarchical models [16]. The statistical heterogeneity among the included studies was quantified using the between-study variances (τ) in this Bayesian meta-analysis, with lower values of τ indicating smaller heterogeneity. Significant heterogeneity was considered substantial if τ exceeded 1.5. The Bayesian forest plots of the meta-analysis provided both study estimates and shrinkage estimates, thereby allowing for a more comprehensive analysis of the pooled effect sizes.

The risk of bias in each included RCTs was evaluated using the revised Cochrane Risk of Bias tool (version 2.0) [17]. In addition, a classic funnel plot was performed to detect any possible publication bias, as it can impact the validity of meta-analyses. All statistical analyses were conducted using the R program with packages “meta,“ “metafor,“ “bayesmeta,“ and “forestplot,“ which were used to extract and analyze the data in this study. The detailed code for the R program was provided in Supplementary File 1.

## Results

### Eligible studies and characteristics

**Fig. 1:**
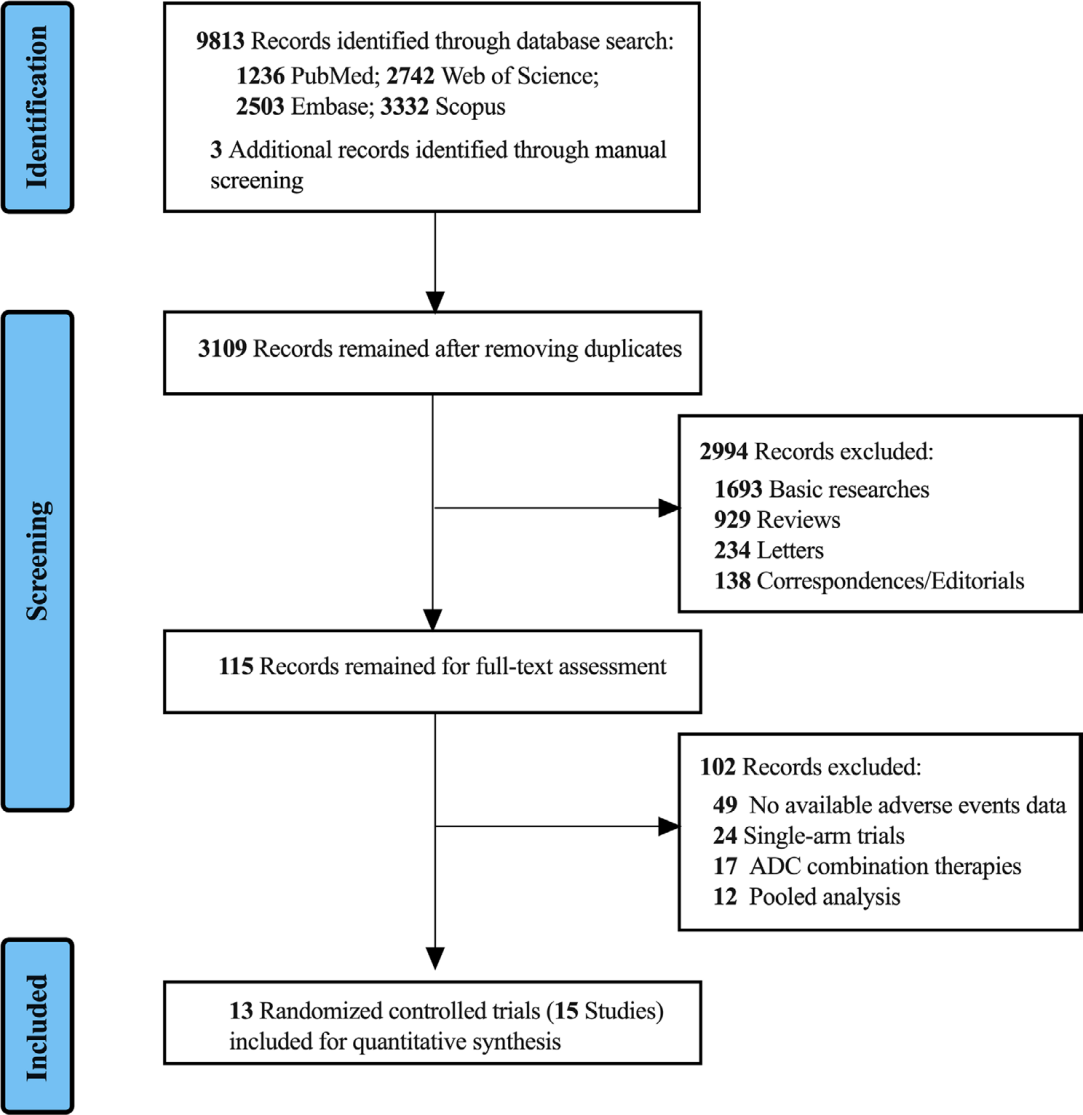
Preferred Reporting Items for Systematic Review and Meta-analysis (PRISMA) flow diagram of included studies

Through a systematic literature search, a total of 9,816 records on HER2-targeted ADCs were obtained from mentioned databases. Following the removal of duplicate references, we then excluded those records about basic research (n = 1,693), review articles (n = 929), correspondences (n = 138), and letters (n = 234), resulting in 115 remaining records. Through full-text article evaluation, 102 records that did not meet the inclusion criteria were excluded, including records without documenting detailed adverse effects (n = 49), ADCs were used in combination with other drugs (n = 17), meta-analysis (n = 12), and single-arm trials (n = 24). Ultimately, we included 15 eligible studies (13 RCTs) [12, 18–27] for quantitative analysis (Fig. 1**)**. These studies represented 12 studies involved in T-DM1 and three studies involved in T-DXd. Of the 15 eligible studies in the systematic review, 13 were conducted in patients with breast cancer and two with gastric cancer. Detailed study characteristics are presented in Table 1.

**Table 1:** Characteristics of included studies assessing fatal adverse events of HER2-targeted drugs in patients with cancer

Author	Year	Study	NCT NO.	Phase	Cancer types	ADC drugs	Control-Arm therapies	Total patients (ADC)	Total patients (Control)	Deaths in ADC	Deaths in Control
Perez	2019	MARIANNE	NCT01120184	3	Breast cancer	T-DM1	Trastuzumab plus taxane	361	353	5	7
Perez	2019	MARIANNE	NCT01120184	3	Breast cancer	T-DM1	T-DM1 plus pertuzumab	361	366	5	7
Krop	2017	TH3RESA	NCT01419197	3	Breast cancer	T-DM1	Physician’s choice chemotherapy	403	184	9	3
Cortés	2022	DESTINY-Breast03	NCT03529110	3	Breast cancer	T-DM1	T-DXd	261	257	0	0
Cortés	2020	TRAXHER2	NCT01702558	2	Breast cancer	T-DM1	T-DM1 plus capecitabine	78	82	0	0
Diéras	2017	EMILIA	NCT00829166	3	Breast cancer	T-DM1	Capecitabine plus lapatinib	490	488	4	5
Minckwitz	2019	KATHERINE	NCT01772472	3	Breast cancer	T-DM1	Trastuzumab	740	720	1	0
Emens	2020	KATE2	NCT02924883	2	Breast cancer	T-DM1	T-DM1 plus atezolizumab	68	132	0	1
Hurvitz	2013	TDM4450g	NCT00679341	2	Breast cancer	T-DM1	Trastuzumab plus docetaxel	69	66	1	1
Tolaney	2021	ATEMPT	NCT01853748	2	Breast cancer	T-DM1	Paclitaxel plus trastuzumab	383	114	0	0
Thungappa	2022	NA	CTRI/2018/07/014881	3	Breast cancer	T-DM1	Biosimilar drug of T-DM1)	55	113	2	6
Thuss-Patience	2017	GATSBY	NCT01641939	2/3	Gastric cancer	T-DM1	Physician’s choice	224	111	8	4
Cortes	2022	DENSTINY-Breast03	NCT03529110	3	Breast cancer	T-DXd	Physician’s choice	257	261	0	0
Shitara	2020	DENSTINY-Gastric01	NCT03329690	2	Gastric cancer	T-DXd	T-DM1	125	62	1	2
Modi	2022	DENSTINY-Breast04	NCT03734029	3	Breast cancer	T-DXd	Physician’s choice	371	172	7	5

*NA: Not applicable, NCT: National Clinical Trial, T-DM1: Trastuzumab emtansine, T-DXd: Trastuzumab deruxtecan*,

A total of 7,727 patients were involved in this meta-analysis. They were randomized in the 15 studies, of which 4,246 patients received HER2-targeted ADCs, while the remaining 3,481 received control treatment. All the patients in these studies had a performance status (PS) between 0 and 2. In the HER2-targeted ADCs arms, 3,453 patients received T-DM1, while T-DXd was used in 753 patients.

### Study quality

The revised Cochrane Risk of Bias tool (RoB version 2.0) was employed for the assessment of the quality of each included study. The quality assessment results indicated that almost entirely of the included RCTs had a low risk of bias except for a high risk of bias found in one study. The detailed assessment results regarding the risk of bias were provided in **eTable 3** in the Supplement.

### Incidence and types of fatal adverse events

**Fig. 2:**
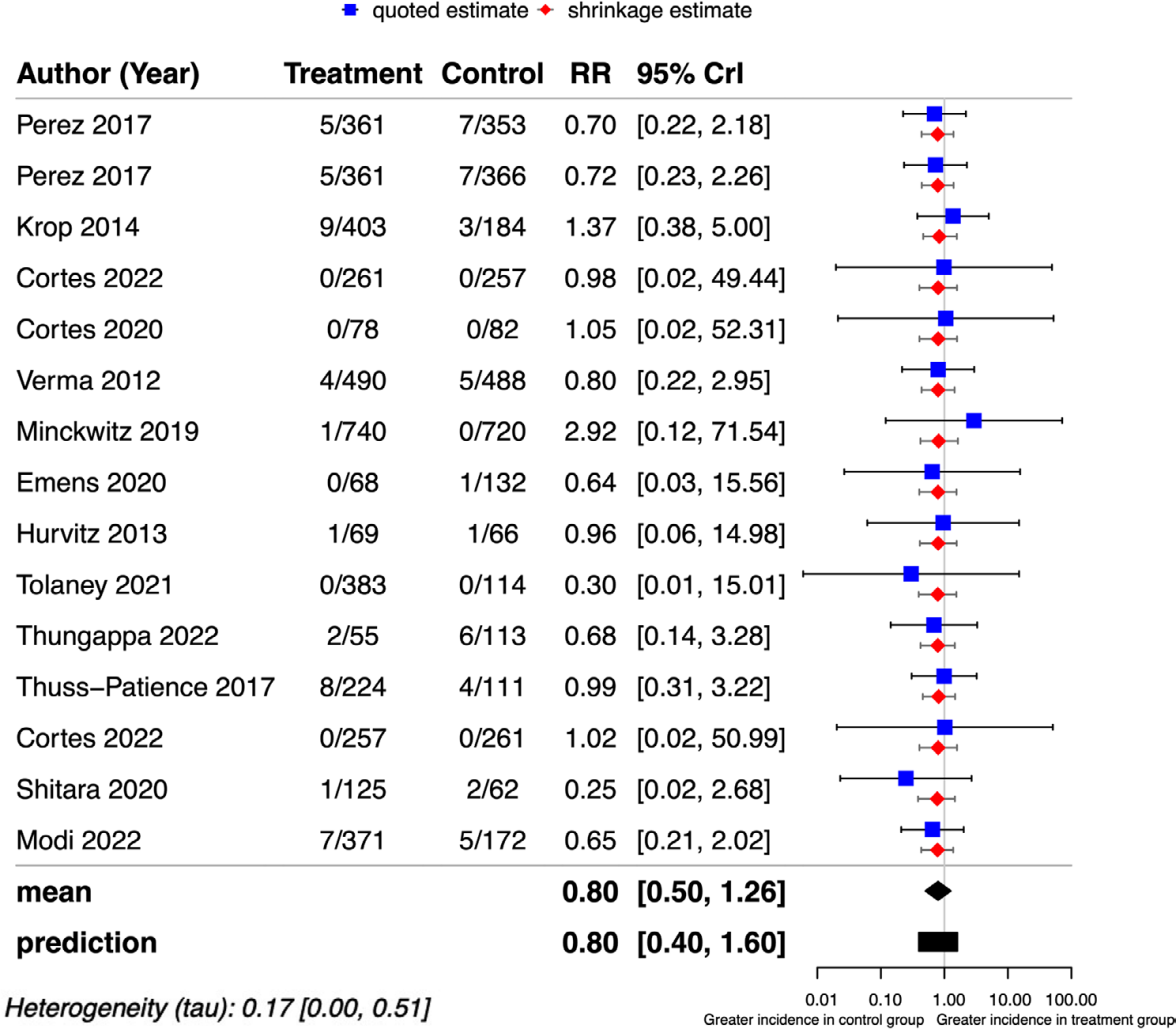
Forest plots of the model posteriors for an overall incidence of mortality caused by HER2-targeted ADCs. Forest plots from the 15 studies display the median and CrI of posterior µ and θ_k+1_ estimates. Quoted study estimates y_i_ and shrinkage estimate θ_i_ for i = 1–15 are also shown. The posterior median µ is shown as incidence, CrI = credible interval

A total of 4,246 patients from 15 studies were included to analyze the incidence of fatal adverse events associated with HER2-targeted ADCs. No fatal adverse events were reported in five studies, and 43 patients experienced fatal adverse events The data were combined using Bayesian hierarchical modeling, which allowed for the estimation of the mean incidence of fatal adverse events to be 0.78% (95% CrI: 0.28-1.37%, τ = 0.006) in the patients treated with HER2-targeted ADC (Fig. 2). The highest incidence (3.64%; 95% CrI, 1.31–8.58%) was observed in a phase III trial of T-DM1 in patients with HER2-positive metastatic breast cancer in India.

From the available data, the most frequently occurring fatal adverse event associated with HER2-targeted ADCs was respiratory toxicity, representing 16 deaths or 37.0% of all study deaths. It includes six pneumonia, three pneumonitis, and one each of interstitial lung disease, pulmonary embolism, pneumonia aspiration, lung infection, bronchopneumonia, dyspnea, and atypical pneumonia (Table 2**)**. Hematologic toxicity was the second most common fatal adverse event caused by HER2-targeted ADCs, representing nine deaths or 20.9% of all deaths. Other less frequent fatal adverse events were infection (n = 6) and hepatic toxicity (n = 2). The cause of the other nine deaths was not known or was not reported.

**Table 2:** The detailed cause of death of HER2-targeted ADCs related death in published clinical trials

Cause of Death	Total deaths (n = 43 in 4246 patients)
Respiratory	n = 16
pneumonia	6
pneumonitis	3
pulmonary embolism	1
interstitial lung disease	1
pneumonia aspiration	1
lung infection	1
bronchopneumonia	1
dyspnea	1
atypical pneumonia	1
**Hematologic**	**n = 9**
febrile neutropenia	2
pulmonary alveolar haemorrhage	1
gastric haemorrhage	1
upper gastrointestinal haemorrhage	1
subarachnoid haemorrhage	1
disseminated intravascular coagulation	1
decreased platelet count	1
acute myeloid leukaemia	1
**Infectious**	**n = 6**
sepsis	3
septic shock	1
neutropenic sepsis	1
ischemic colitis	1
**Hepatic**	**n = 2**
hepatic dysfunction	1
hepatic encephalopathy	1
**Others**	**n = 1**
Metabolic encephalopathy	1
**Unspecific**	**n = 9**
Sudden death	1
Death of unknown cause	1
Not mentioned	7

### Relative risk of fatal adverse events and subgroup analysis

**Fig. 3:**
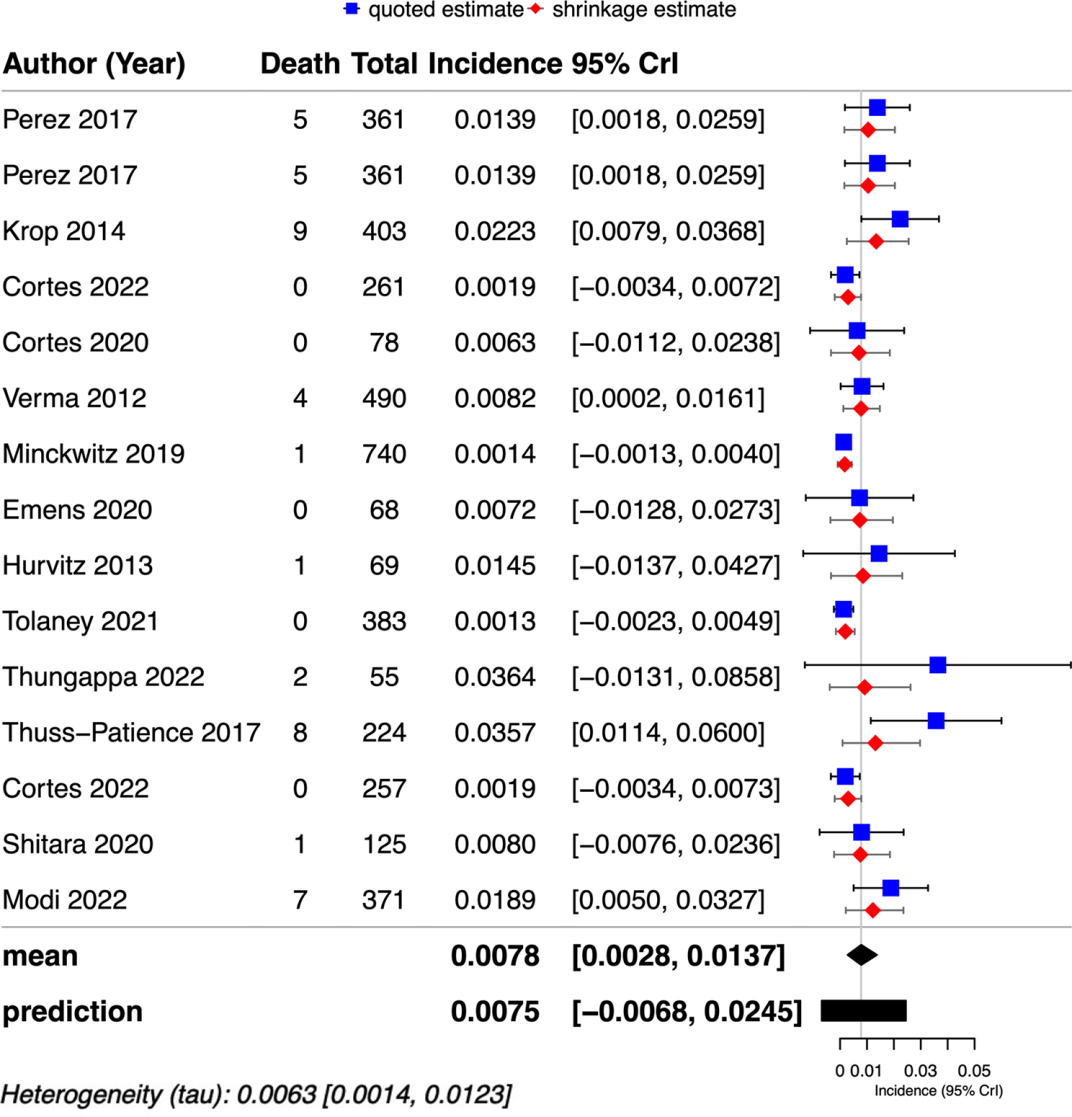
Forest plots of the model posteriors for overall risk of mortality caused by HER2-targeted ADCs. Forest plots from the 15 studies display the median and CrI of posterior µ and θ_k+1_ estimates. Quoted study estimates y_i_ and shrinkage estimate θ_i_ for i = 1–15 are also shown. The posterior median µ is shown as exponentiated (standard) linear risk ratios, wherein a null effect equals 1. CrI = credible interval

**Fig. 4:**
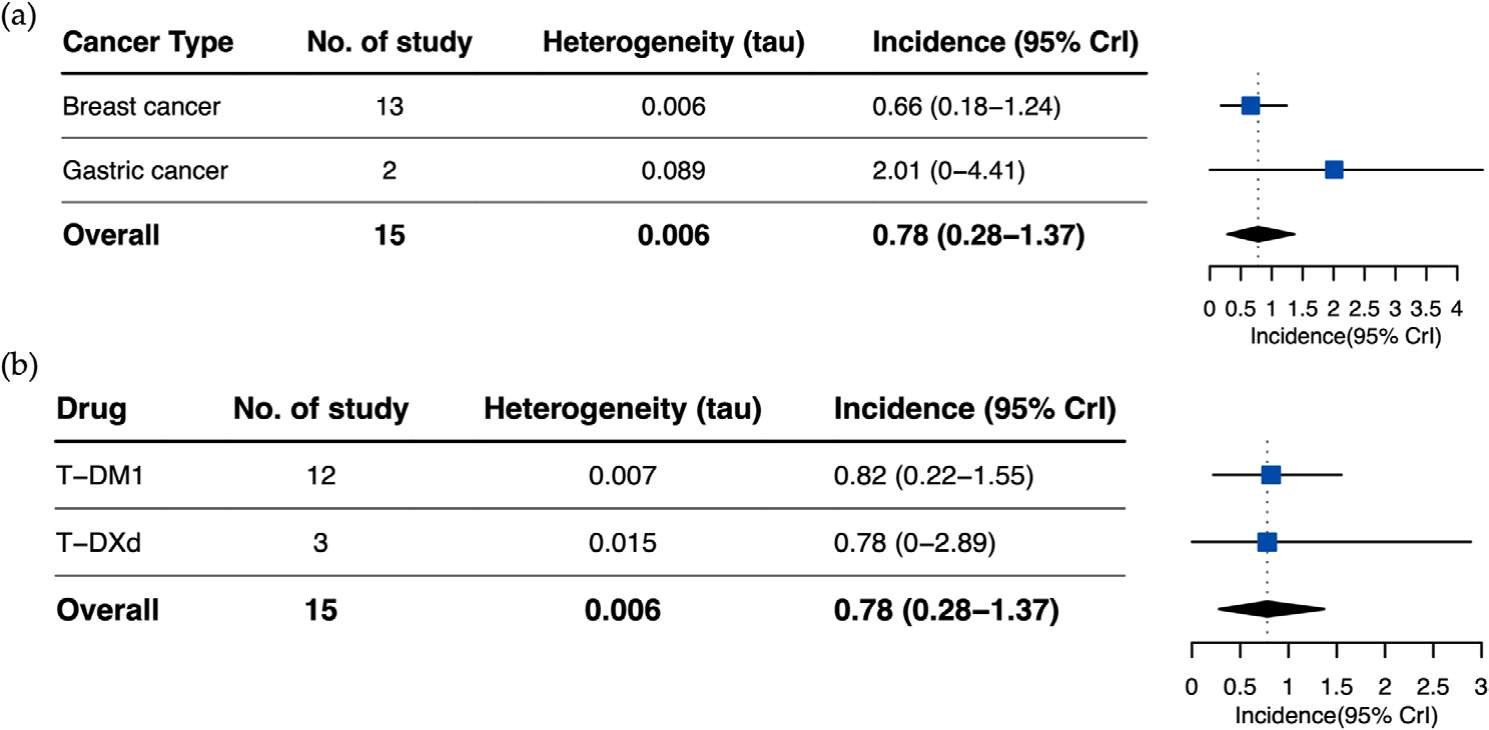
Subgroup analysis of fatal adverse events of HER2-targeted ADCs. (A) Incidence of fatal adverse events related to HER2-targeted ADCs by the cancer types. (B) Incidence of fatal adverse events related to HER2-targeted ADCs by drugs

The pooled incidence of fatal adverse events in patients who received HER2-targeted ADCs was 0.078% (43/4,246) versus 0.095% (41/3,481) in patients in the control arm. The summary relative risk for developing fatal adverse events with the HER2-targeted ADCs across the studies was RR = 0.80 (95% CrI, 0.5–1.26, τ = 0.17) (Fig. 3). When stratified by each used HER2-targeted ADC, the incidence was 0.82% (95% CrI, 0.22–1.55%, τ = 0.007) for T-DM1 and 0.78%(95%CrI,0–2.89%, τ = 0.015) for T-DXd. As for the different cancer types, the incidences of the fatal adverse event caused by HER2-targeted ADCs in breast cancer patients and gastric cancer patients were determined as 0.66% (95% CrI, 0.18–1.24%, τ = 0.006) and 2.01%(95%CrI,0–4.41%, τ = 0.089), respectively. The subgroup analyses based on HER2-targeted ADC drugs and cancer types did not reveal any meaningful differences. Figure 4 demonstrates the overall and stratified analysis.

### Sensitivity analysis and publication bias

We conducted a sensitivity analysis by using various prior distributions for the between-study variance (τ) and presented the results in **eTable 4** in the Supplement. The sensitivity analysis indicated that the RR of fatal adverse events with HER2-targeted ADCs remained consistent, supporting the robustness of our estimated effect size using Bayesian hierarchical modeling. Additionally, we assessed the possibility of publication bias using a classic funnel plot [28]. The funnel plot (**eFigure 1** in Supplement) indicated significant asymmetry in fatal adverse events, indicating no publication bias in the included trials.

## Discussion

Based on the 13 clinical RCTs, including 4,246 patients, the pooled incidence of fatal adverse events in patients treated with a HER2-targeted ADC was 0.078% compared with 0.095% in patients from control arms, and this risk was lower but not significantly than that with the control arm (RR = 0.8; 95% CrI, 0.5–1.26, τ = 0.17). These findings suggest that HER2-targeted ADCs may be a relatively safe and comparable alternative to standard conventional therapies for cancer patients, making them promising novel therapeutic options in clinical settings.

As an emerging biopharmaceutical drug, the HER2-targeted ADCs have provided promising alternative ways to fight against cancer [29]. However, the information on fatal adverse events associated with HER2-targeted ADCs remains unclear. Cancer therapy using HER2-targeted ADCs is a double-edged sword. While focusing on efficacy, we should also pay close attention to the adverse event caused by drugs, especially the fatal ones, since it severely impacts patients and their families. Therefore, it is necessary to investigate the incidence of fatal adverse events to properly evaluate the benefit-risk ratio and make decisions in the oncology clinic. Our study showed that treatment with HER2-targeted ADCs results in 0.078% of patients dying due to adverse effects of ADC treatment alone. It also revealed a lower risk of fatal adverse events compared to the other standard therapies (RR = 0.8). These data should be essential in considering whether to use HER2-targeted ADCs treatment.

Among 43 reported deaths, the most common cause of death caused by HER2-targeted ADCs was respiratory toxicity, including six pneumonia, three pneumonitis, and one each of interstitial lung disease, pulmonary embolism, pneumonia aspiration, lung infection, bronchopneumonia, dyspnea, and atypical pneumonia. Besides, hematologic toxicity, infection, and hepatic toxicity accounted for the other leading cause of death. This meta-analysis demonstrates that the risk of fatal adverse events with HER2-targeted ADCs is comparable to conventional anticancer therapy. Therefore, it is crucial to closely monitor patients receiving HER2-targeted ADCs for symptoms related to the respiratory system, infection, and liver functions. Early recognition and management of toxic effects, including prompt initiation of dose reduction and other modulating agents like glucocorticoids, are essential for preventing fatalities.

Previous meta-analyses have estimated the incidence and risk of adverse events associated with T-DM1, but they did not analyze fatal events [30–33]. There was also a meta-analysis that investigated the incidence of general adverse events related to antibody-drug conjugates in all clinical trials, including lymphopenia, nausea, neutropenia, peripheral neuropathy, and blurred vision [34]. However, our study concentrated on fatal adverse events instead of general adverse events and also focused on the HER2-targeted ADCs, which represent the most common subclass of ADCs. Our meta-analysis is the largest to date, including 4,246 patients from high-quality RCTs, and provides a summary of HER2-targeted ADCs-related fatal adverse events in cancer patients. The results revealed that the toxicities of the respiratory system and myelosuppression attributed to the leading cause of death among patients receiving HER2-targeted ADCs. The reasonable interpretation of that was the high amounts of FcγR expressed in alveolar macrophages and myeloid cells and Fc-mediated non-specific uptake of HER2-targeted ADCs might contribute to these fatal adverse events [35, 36]. Consequently, the next generation of HER2-targeted ADCs could consider optimizing the Fc fragments of the antibody part of ADCs, such as an increase in serum stability or improvement of binding specificity and affinity.

However, some limitations could be improved in our study. Firstly, our study relied on study-level data, and individual patient-level confounding factors could not be thoroughly assessed or included in the analysis. Secondly, since the primary outcomes of included RCTs were focused on the efficacy of HER2-targeted ADCs, the fatal adverse events were reported through different investigators and institutions, which might introduce potential bias in the assessment of whether fatal adverse events were associated with the treatment of HER2-targeted ADCs. Thirdly, probably because of the small sample size and potential reasons related to cancer, our analysis showed no evident risk difference between HER2-targeted ADCs and control-arm therapies. Finally, due to the scarcity of studies involving T-DXd in non-breast cancer patients, the pre-defined stratification factors (i.e., drug and cancer types) were insufficient to detect significant differences in the risk associated with distinct HER2-ADCs or cancer types.

## Conclusions

Based on our systematic review and Bayesian meta-analysis, this study reveals the incidence and risk of fatal adverse events associated with HER2-targeted ADCs in cancer patients involved in RCTs. The results indicate that the risk of fatal adverse events with HER2-targeted ADCs may be lower compared to standard control therapies in cancer patients. Moreover, our study found no significant difference in the risk of fatal adverse events between different HER2-targeted ADCs or cancer types. However, the most common fatal adverse event was respiratory toxicity, suggesting that cancer patients who use the above drugs should strengthen respiratory system monitoring and take preventive measures in some severe cases.

### Electronic supplementary material

Below is the link to the electronic supplementary material.

Supplementary Material 1

## Data Availability

All data generated or analyzed during this study are included in this published article [and its supplementary information files.
