# Supplementary material for: Incidence and risk of fatal adverse events in cancer patients treated with HER2-targeted antibody-drug conjugates: a systematic review and meta-analysis of randomized controlled trials
Source: BMC Cancer. 2023 Oct 10;23:960. doi: 10.1186/s12885-023-11250-1 (PMC10563201; doi:10.1186/s12885-023-11250-1)
Supplement: Supplementary file 1 — Supplementary Material 1 [file 12885_2023_11250_MOESM1_ESM.docx]

**Supplement**

**Supplementary file 1: Bayesian hierarchical model and the code from R software**

**1. The Bayesian hierarchical model**

Meta-analysis usually combines aggregated or individual results from several studies to create a pooled, more precise estimate of an effect. Due to the hierarchical structure (intra-study and between-study) of meta-analysis data, the Bayesian hierarchical approach is often used in meta-analysis ^1,2^. Meanwhile, the Bayesian approach allows us to account for uncertainty from the varying quality of data and borrow strength from non-missing data, and MCMC sampling allows for inference in a high-dimensional, constrained parameter space, while providing posterior estimation that allow straightforward inference on the wide variety of functionals of interest. In our meta-analysis, the outcomes of interest were the incidences of adverse events associated with HER2-targeted antibody-drug conjugates in clinical trials. We use the Bayesian Hierarchical models to estimate the pooled incidences of all-grade adverse events, high-grade (grade 3 or higher) adverse events, serious adverse events, and adverse events that resulted in drug discontinuation.

For the *i*th study which reported the dichotomous outcomes, the number of patients with reported any adverse events in the *i*th study followed the binomial distribution:

*r_i_* ~binomial (𝑛_𝑖_ , 𝑝_𝑖_) (1)

Where 𝑛_𝑖_ was the total number of investigated population and 𝑝_𝑖_ was the incidence of adverse event for the *i*th study.

The logit transformation of 𝑝_𝑖_ followed a normal distribution among studies:

𝜃_𝑖_ = logit( 𝑝_𝑖_ )~normal (𝜇, 𝜎^2^ ) (2)

Where 𝜇 was the mean of logit(𝑝_𝑖_ ) and 𝜎^2^ was the between-study variance.

Then we could estimate the pooled incidence of adverse event and the corresponding 95% credible interval (CrI) through retransform the

*Incidence* = exp (𝜇)/(1 + 𝑒𝑥𝑝 (𝜇)) (3)

**2. Parameter Estimation**

The Bayesian random effects model was applied to generate the estimates of the overall incidence and relative risk compared to the control arm, along with a 95% credible interval (CrI). The CrI represented the 2.5-97.5 percentiles of the posterior distribution of the estimation. For the mean parameters of normal distributions, a proper prior distribution with mean=0 and sd=4 was proposed. The between-study variances were assigned weakly informative normal prior distributions with the mode at 0 and the scale at 1. The posterior distribution of interest outcomes was estimated using the Markov Chain Monte Carlo (MCMC) algorithm and Gibbs sampling in all Bayesian hierarchical models. The statistical heterogeneity among the included studies was quantified using the between-study variances (τ) in this Bayesian meta-analysis, with lower values of τ indicating smaller heterogeneity. Significant heterogeneity was considered substantial if τ exceeded 1.5. The Bayesian forest plots of the meta-analysis provided both study estimates and shrinkage estimates, thereby allowing for a more comprehensive analysis of the pooled effect sizes.

**3. Code of “*bayesmeta*” package from R software**

set.seed(12345)

*library(metafor)*

*library(meta)*

*library(bayesmeta)*

*setwd("~/Downloads/Fatal AE-Meta/")*

*data=read.csv("~/Downloads/Fatal AE-Meta/data_bayes.csv", header=T, sep=",")*

*crins.es <- escalc(measure = "RR", ai = r1,bi=m1,ci = r2,di=m2, n1i=n1,n2i=n2,slab = data$authoryear,data = data)*

*crins.es*

*ma01 <- bayesmeta(y = crins.es[, "yi"], sigma = sqrt(crins.es[, "vi"]),*

*labels = crins.es[, "authoryear"], mu.prior.mean = 0, mu.prior.sd = 4,*

*tau.prior = function(t) dhalfnormal(t, scale = 1))*

*summary(ma01)*

*fp <- forestplot(ma01, expo=TRUE, plot=FALSE)*

*labtext <- fp$labeltext*

*labtext[1,1] <- "Author (Year)"*

*labtext <- cbind(labtext[,1],*

*c("Treatment",*

*paste0(crins.es[,"r1"], "/", crins.es[,"n1"]),*

*"",""),*

*c("Control",*

*paste0(crins.es[,"r2"], "/", crins.es[,"n2"]),*

*"",""),*

*labtext[,2:3])*

*labtext[1,4] <- "RR"*

*labtext[1,5] <- "95% CrI"*

*print(fp$labeltext) # before*

*print(labtext) # after*

*ma01*

*forestplot(ma01, labeltext=labtext, expo=TRUE,xticks=c(0.01,0.1,1,10,100),xticks.digit=0,xlog=TRUE,*

*graph.pos=6,line.margin=0.1, ci.vertices = TRUE,*

*ci.vertices.height = 0.05,*

*align = c("l","c","c", "c", "l"),boxsize=0.4,zero=1,*

*xlab=" Greater incidence in control group Greater incidence in treatment group", title="Risk of fatal adverse events with HER2-ADCs",*

*col=fpColors(box = c("blue", "red"),summary= c("black","violet"),lines=c("black", "gray50")),*

*lineheight = unit(0.08, "npc"),*

*graphwidth = unit(0.2, "npc"),*

*colgap = unit(0.02,"npc"),*

*txt_gp = fpTxtGp(label=gpar(cex=0.9),ticks = gpar(cex=0.6), xlab = gpar(cex=0.5)))*

*ma01$summary*

*jag.sum<-ma01$summary*

*write.table(x=jag.sum,file="RR.txt",sep="\t")*

*###publication bias*

*funnel.bayesmeta(ma01,xlab = "lnRR")*

*####Incidence*

*ies.ft=escalc(xi=r1, ni=n1, data=data, measure ="PR",add=0.5,slab = data$authoryear)*

*ies.ft*

*bma12 <- bayesmeta(ies.ft,*

*mu.prior.mean = 0, mu.prior.sd = 10, labels = ies.ft[, "authoryear"],*

*tau.prior = function(t) dhalfnormal(t, scale = 1))*

*summary(bma12)*

*fp <- forestplot(bma12, expo=FALSE, plot=FALSE)*

*labtext <- fp$labeltext*

*labtext[1,1] <- "Author (Year)"*

*labtext <- cbind(labtext[,1],*

*c("Death",*

*paste0(ies.ft[,"r1"]),*

*"",""),*

*c("Total",*

*paste0(ies.ft[,"n1"]),*

*"",""),*

*labtext[,2:3])*

*labtext[1,4] <- "Incidence"*

*labtext[1,5] <- "95% CrI"*

*print(fp$labeltext) # before*

*print(labtext) # after*

*forestplot(bma12, labeltext=labtext, expo=FALSE,xticks=c(0,0.01,0.02,0.03,0.04,0.05),xticks.digit=4,*

*graph.pos=6,line.margin=0.1, ci.vertices = TRUE,*

*ci.vertices.height = 0.05,*

*align = c("l","c","c", "c", "l"),boxsize=0.4,zero=0.0078,*

*xlab=" Incidence (95% CrI)",*

*col=fpColors(box = c("blue", "red"),summary= c("black","violet"),lines=c("black", "gray50")),*

*lineheight = unit(0.08, "npc"),*

*graphwidth = unit(0.2, "npc"),*

*colgap = unit(0.02,"npc"),*

*txt_gp = fpTxtGp(label=gpar(cex=0.9),ticks = gpar(cex=0.6), xlab = gpar(cex=0.5)))*

**eTable 1: PRISMA checklist^3^**

| **Section/topic** | **#** | **Checklist item** | **Reported on page #** |
| --- | --- | --- | --- |
| **TITLE** | | |  |
| Title | 1 | Identify the report as a systematic review, meta-analysis, or both. | 1 |
| **ABSTRACT** | | |  |
| Structured summary | 2 | Provide a structured summary including, as applicable: background; objectives; data sources; study eligibility criteria, participants, and interventions; study appraisal and synthesis methods; results; limitations; conclusions and implications of key findings; systematic review registration number. | 2-3 |
| **INTRODUCTION** | | |  |
| Rationale | 3 | Describe the rationale for the review in the context of what is already known. | 4 |
| Objectives | 4 | Provide an explicit statement of questions being addressed with reference to participants, interventions, comparisons, outcomes, and study design (PICOS). | 5 |
| **METHODS** | | |  |
| Protocol and registration | 5 | Indicate if a review protocol exists, if and where it can be accessed (e.g., Web address), and, if available, provide registration information including registration number. | 5 |
| Eligibility criteria | 6 | Specify study characteristics (e.g., PICOS, length of follow-up) and report characteristics (e.g., years considered, language, publication status) used as criteria for eligibility, giving rationale. | 5 |
| Information sources | 7 | Describe all information sources (e.g., databases with dates of coverage, contact with study authors to identify additional studies) in the search and date last searched. | 5 |
| Search | 8 | Present full electronic search strategy for at least one database, including any limits used, such that it could be repeated. | 5 |
| Study selection | 9 | State the process for selecting studies (i.e., screening, eligibility, included in systematic review, and, if applicable, included in the meta-analysis). | 5-6 |
| Data collection process | 10 | Describe method of data extraction from reports (e.g., piloted forms, independently, in duplicate) and any processes for obtaining and confirming data from investigators. | 6 |
| Data items | 11 | List and define all variables for which data were sought (e.g., PICOS, funding sources) and any assumptions and simplifications made. | 6 |
| Risk of bias in individual studies | 12 | Describe methods used for assessing risk of bias of individual studies (including specification of whether this was done at the study or outcome level), and how this information is to be used in any data synthesis. | 6 |
| Summary measures | 13 | State the principal summary measures (e.g., risk ratio, difference in means). | 6-7 |
| Synthesis of results | 14 | Describe the methods of handling data and combining results of studies, if done, including measures of consistency (e.g., I^2^) for each meta-analysis. | 6-7 |

| **Section/topic** | **#** | **Checklist item** | **Reported on page #** |
| --- | --- | --- | --- |
| Risk of bias across studies | 15 | Specify any assessment of risk of bias that may affect the cumulative evidence (e.g., publication bias, selective reporting within studies). | 7 |
| Additional analyses | 16 | Describe methods of additional analyses (e.g., sensitivity or subgroup analyses, meta-regression), if done, indicating which were pre-specified. | 7 |
| **RESULTS** | | |  |
| Study selection | 17 | Give numbers of studies screened, assessed for eligibility, and included in the review, with reasons for exclusions at each stage, ideally with a flow diagram. | 7 |
| Study characteristics | 18 | For each study, present characteristics for which data were extracted (e.g., study size, PICOS, follow-up period) and provide the citations. | 8 |
| Risk of bias within studies | 19 | Present data on risk of bias of each study and, if available, any outcome level assessment (see item 12). | 8 |
| Results of individual studies | 20 | For all outcomes considered (benefits or harms), present, for each study: (a) simple summary data for each intervention group (b) effect estimates and confidence intervals, ideally with a forest plot. | 8 |
| Synthesis of results | 21 | Present results of each meta-analysis done, including confidence intervals and measures of consistency. | 8-9 |
| Risk of bias across studies | 22 | Present results of any assessment of risk of bias across studies (see Item 15). | 9 |
| Additional analysis | 23 | Give results of additional analyses, if done (e.g., sensitivity or subgroup analyses, meta-regression [see Item 16]). | 9 |
| **DISCUSSION** | | |  |
| Summary of evidence | 24 | Summarize the main findings including the strength of evidence for each main outcome; consider their relevance to key groups (e.g., healthcare providers, users, and policy makers). | 10-12 |
| Limitations | 25 | Discuss limitations at study and outcome level (e.g., risk of bias), and at review-level (e.g., incomplete retrieval of identified research, reporting bias). | 12 |
| Conclusions | 26 | Provide a general interpretation of the results in the context of other evidence, and implications for future research. | 12 |
| **FUNDING** | | |  |
| Funding | 27 | Describe sources of funding for the systematic review and other support (e.g., supply of data); role of funders for the systematic review. | 13 |

**eTable 2: Search strategy (Access Date: 1**4 August 2022**)**

| **PubMed (Search Results:** 2742**)** |
| --- |
| **Search strategy:** Ado Trastuzumab Emtansine[Title/Abstract] OR Trastuzumab Emtansine[Title/Abstract] OR Kadcyla[Title/Abstract] OR Trastuzumab DM1 Conjugate[Title/Abstract] OR Trastuzumab DM1[Title/Abstract] OR T-DM1[Title/Abstract] OR trastuzumab deruxtecan[Title/Abstract] OR DS-8201[Title/Abstract] OR DS-8201a[Title/Abstract] OR T-Dxd[Title/Abstract]  **Search Results:** 1236 |
| **Web of Science (Search Results:** 2742**)** |
| **Search strategy:** TS= (Ado Trastuzumab Emtansine OR Trastuzumab Emtansine OR Kadcyla OR Trastuzumab DM1 OR T-DM1 OR Trastuzumab DM1 Conjugate OR trastuzumab deruxtecan OR DS-8201 OR DS-8201a OR T-Dxd) |
| **Embase (Search Results:** 2503**)** |
| **Search strategy:** 'Ado Trastuzumab Emtansine':ab,ti OR 'Trastuzumab Emtansine':ab,ti OR 'Kadcyla':ab,ti OR 'Trastuzumab DM1 Conjugate':ab,ti OR 'Trastuzumab DM1':ab,ti OR 'T-DM1':ab,ti OR 'trastuzumab deruxtecan':ab,ti OR 'DS-8201':ab,ti OR 'DS-8201a':ab,ti OR 'T-Dxd':ab,ti |
| **Scopus (Search Results:** 3332**)** |
| **Search strategy:** TITLE-ABS-KEY("Ado Trastuzumab Emtansine" OR "Trastuzumab Emtansine" OR "Kadcyla" OR "T-DM1" OR "Trastuzumab DM1" OR "Trastuzumab DM1 Conjugate" OR "trastuzumab deruxtecan" OR "DS-8201" OR "DS-8201a" OR "T-Dxd") |

**eTable 3. The RoB-2 tool^4^ for assessing the quality of included Randomized Controlled Trials (n=13)**

| Study | Randomization | Allocation concealment | Blinding of participants and staff | Blinding of outcome assessors | Incomplete outcome data | Selective reporting | Other bias | Overall bias |
| --- | --- | --- | --- | --- | --- | --- | --- | --- |
| Cortés 2022 | Low | Low | Low | Low | Low | Low | Low | Low |
| Cortés 2020 | Low | Low | Low | Low | Low | Low | Low | Low |
| Emens 2020 | Low | Low | Low | Low | Low | Low | Low | Low |
| Hurvitz 2013 | Low | Low | Low | Low | Low | Low | Low | Low |
| Krop 2017 | Low | Low | Low | Low | Low | Low | Low | Low |
| Minckwitz 2019 | Low | Low | Low | Low | Low | Low | Low | Low |
| Modi 2022 | Low | Low | Low | Low | Low | Low | Low | Low |
| Perez 2019 | Low | Low | Low | Low | Low | Low | Low | Low |
| Shitara 2020 | Low | Low | Low | Low | Low | Low | Low | Low |
| Thungappa 2022 | Low | Some concerns | Some concerns | Some concerns | Low | Low | Some concerns | High |
| Thuss-Patience 2017 | Low | Low | Low | Low | Low | Low | Low | Low |
| Tolaney 2021 | Low | Low | Low | Low | Low | Low | Low | Low |
| Verma 2012 | Low | Low | Low | Low | Low | Low | Low | Low |

Rob-2: Version 2 of the Cochrane risk-of-bias tool for randomized trials

**eTable 4.** Estimated relative risk (lnRR) of fatal adverse events associated with HER2-targeted ADCs for sensitivity analysis

| Models | Prior distribution for the mean (μ_i_) | Prior distribution for the variance (τ) | Posterior mean µ (ln RR, 95% CrI) | Posterior variance τ (95% CrI) |
| --- | --- | --- | --- | --- |
| 1 (used in this study) | Normal (mean=0, sd=4) | dhalfnormal (t, scale = 0.5) | -0.22 (-0.68, 0.23) | 0.17 (0-0.50) |
| 2 | Normal (mean=0, sd=10) | dhalfnormal (t, scale = 0.5) | -0.23 (-0.69, 0.23) | 0.17 (0-0.50) |
| 3 | Normal (mean=0, sd=4) | dhalfnormal (t, scale = 1.0) | -0.23 (-0.69, 0.24) | 0.18 (0-0.50) |
| 4 | Normal (mean=0, sd=4) | dhalfcauchy (t, scale = 0.5) | -0.23 (-0.68, 0.23) | 0.16 (0-0.50) |
| 5 | Normal (mean=0, sd=4) | uniform | -0.23 (-0.70, 0.27) | 0.19 (0-0.63) |

*CrI: credible interval; τ: between-study variance which used to indicate heterogeneity*

**eFigure 1. Funnel plot of the relative risk of fatal adverse events associated with HER2-targeted ADCs**

**References**

1. Liu B, Lahiri P, Kalton G. Hierarchical Bayes modeling of survey-weighted small area proportions. Paper presented at: Proceedings of the American Statistical Association, Survey Research Section2007.

2. Vasudev D, Goswami VR. A Bayesian hierarchical approach to quantifying stakeholder attitudes toward conservation in the presence of reporting error. *Conservation Biology.* 2020;34(2):515-526.

3. Page MJ, McKenzie JE, Bossuyt PM, et al. The PRISMA 2020 statement: an updated guideline for reporting systematic reviews. *Systematic reviews.* 2021;10(1):1-11.

4. Sterne JA, Savović J, Page MJ, et al. RoB 2: a revised tool for assessing risk of bias in randomised trials. *bmj.* 2019;366.
